# The Mechanisms of Ferroptosis and the Applications in Tumor Treatment: Enemies or Friends?

**DOI:** 10.3389/fmolb.2022.938677

**Published:** 2022-07-15

**Authors:** Shuzheng Tan, Ying Kong, Yongtong Xian, Pengbo Gao, Yue Xu, Chuzhong Wei, Peixu Lin, Weilong Ye, Zesong Li, Xiao Zhu

**Affiliations:** ^1^ School of Laboratory Medicine and Biological Engineering, Hangzhou Medical College, Hangzhou, China; ^2^ Department of Dermatology, The First Affiliated Hospital of Guangzhou Medical University, Guangzhou, China; ^3^ Department of Clinical Laboratory, Hubei No.3 People’s Hospital of Jianghan University, Wuhan, China; ^4^ Computational Oncology Laboratory, Guangdong Medical University, Zhanjiang, China; ^5^ Guangdong Provincial Key Laboratory of Systems Biology and Synthetic Biology for Urogenital Tumors, Shenzhen Key Laboratory of Genitourinary Tumor, Department of Urology, The First Affiliated Hospital of Shenzhen University, Shenzhen Second People’s Hospital (Shenzhen Institute of Translational Medicine), Shenzhen, China

**Keywords:** ferroptosis, immunotherapy, iron overload, lipid peroxidation, mitochondria

## Abstract

Ferroptosis, as a newly discovered non-apoptotic cell death mode, is beginning to be explored in different cancer. The particularity of ferroptosis lies in the accumulation of iron dependence and lipid peroxides, and it is different from the classical cell death modes such as apoptosis and necrosis in terms of action mode, biochemical characteristics, and genetics. The mechanism of ferroptosis can be divided into many different pathways, so it is particularly important to identify the key sites of ferroptosis in the disease. Herein, based on ferroptosis, we analyze the main pathways in detail. More importantly, ferroptosis is linked to the development of different systems of the tumor, providing personalized plans for the examination, treatment, and prognosis of cancer patients. Although some mechanisms and side effects of ferroptosis still need to be studied, it is still a promising method for cancer treatment.

## Introduction

Ferroptosis is a kind of iron-dependent programmed cell death, which is different from traditional cell necrosis, apoptosis, and autophagy in morphology, biochemistry, and genetics. It is a new type of programmed cell death. In a word, the mode of cell death in which extensive lipid peroxidation occurs is called ferroptosis (Chen et al., 2021b; Ding et al., 2021).

When Erastin is used to selectively act on the RAL gene in human cancer cells, it is found that lots of iron ions and lipid oxidizing substances were produced in the cells and iron ions catalyzed the oxidation of lipids. Abnormal metabolism of substances leads to cell death on account of destroying the normal redox environment in the cell. Therefore, the essence of ferroptosis is the oxidative death of cells caused by the accumulation of several iron ions. Biologically, the pivotal feature of the ferroptosis is the iron-dependent lipid reactive oxygen accumulation and the activation of the mitogen-activated protein kinase system (Dixon et al., 2012). Morphologically, once ferroptosis occurs, the nucleus is normal in size but lacks chromatin agglutination. At the same time, the mitochondria shrink and the mitochondrial ridges are reduced or even disappear. The outer membrane is broken, and the bilateral membrane density increases in the mitochondria. The application of iron chelating agents and antioxidants can effectively play inhibitory effects on the occurrence of ferroptosis. On the contrary, the supplementation of iron will aggravate this process. Genetically, ferroptosis is primarily regulated by iron response element-binding protein (IREB2), citrate synthase (CS), and ATP synthase F0 complex subunit C3 (ATP5G3). Immunologically, inflammatory mediators released by DAMPs, such as high mobility group protein b1, cause both innate and adaptive immune responses.

Ferroptosis was initially discovered and established only in tumor cells, but as research continues to deepen, ferroptosis is shown to play a vital role in pathological processes such as tumors, neurodegenerative diseases, and tissue ischemia-reperfusion injury (Stockwell et al., 2017).

## Biological Characteristics of Ferroptosis

### Iron Overload

Excessive accumulation of iron is the necessary condition and main characteristic of ferroptosis. Fe^2+^, whose quantity is the kernel to the formation of PL-OOH, serves as the dominating form of iron in the intracellular labile iron pool (LIP). LIP is regulated by intracellular iron homeostasis. Accumulation of PL-OOH is a sign of ferroptosis. The free radicals and hyperoxides generated after the redox reaction of Fe^2+^ and Fe^3+^ can react with Polyunsaturated fatty acids (PUFAs) containing phospholipids (PLs) in the cell membrane to facilitate the spread of lipid peroxidation on the cell membrane. This process produces lots of ROS and induces cell ferroptosis (Doll and Conrad, 2017; Ruiz-de-Angulo et al., 2020; Nieto-Garai et al., 2022). In addition, iron-dependent lipid peroxidation can be attenuated by GPX4, radical trapping protein removal, ferroptosis-specific inhibitors, and iron chelation. William et al. lately found that if iron chelation, such as Deferoxamine (DFO), is added to cells, the occurrence of ferroptosis could be inhibited. In contrast, iron supplements intensify the process (Abrams et al., 2016). This discovery fully reveals that ferroptosis is dependent on iron.

### Lipid Peroxidation

Cell peroxidation caused by lipid reactive oxygen accumulation is the direct cause of ferroptosis (Chu et al., 2019), among which the key lipid is sn2-15-HPET-PE (Anthonymuthu et al., 2018). When lipid antioxidants are applied to cell membranes, lipid degradation could significantly reduce the occurrence of cell ferroptosis (Yang et al., 2016; Doll et al., 2019; Lin Z. et al., 2021). In brief, lipid metabolism is an important process of ferroptosis. Researchers discovered that in addition to long-chain polyunsaturated fatty acid, long-chain saturated fatty acid can potentiate ferroptosis through peroxisome-driven ether phospholipid biosynthesis (Hwang et al., 2021). FAR1 and 1-hexadecanol (1-HE) remarkably accelerate ferroptosis in tumor cells. Moreover, TMEM189 can replace the role of the FAR1-alkyl-ether lipids axis in inducing ferroptosis, which lays the ground for becoming a target of a new generation of anticancer drugs (Cui et al., 2021).

The enzymes that stimulate ferroptosis are the oxidoreductases POR and CYB5R1 located on the endoplasmic reticulum. It was found that phospholipids containing long-chain unsaturated fatty acids are catalyzed by POR and CYB5R1 to produce lipid peroxidation, resulting in oxidative damage of liposome membranes. This process revealed the biochemical mechanism of cell membrane oxidative damage during ferroptosis (Yan et al., 2021). In addition, MDM2 and MDMX promote ferroptosis by regulating PPARα-mediated lipid homeostasis, which is independent of P53. Therefore, MDM2 and MDMX inhibitors can be used to treat diseases associated with ferroptosis (Venkatesh et al., 2020).

### The Role of Mitochondria

Iron and cysteine are involved in the regulation of one of the mechanisms of ferroptosis. Cysteine deficiency results in mitochondrial membrane potential hyperpolarization and lipid peroxides accumulation. But when cysteine is deficient, serum transferrin and glutamine are requisite for ferroptosis. Also, Mitochondria are of the essence in regulating cysteine deprivation-induced (CDI) ferroptosis, including the mitochondrial TCA cycle and mitochondrial electron transport chain (Jiang X. et al., 2021; Wei et al., 2022). In the mitochondrial TCA cycle, the breakdown of glutamine produces α -ketoglutarate to provide energy, thus improving mitochondrial respiration rate and promoting ROS production. Conversely, loss of function of fumarate hydra-tase (FH), a mitochondrial tumor suppressor, causes kidney cancer cells to resist ferroptosis. Moreover, The hyperpolarization of mitochondrial membrane potential promotes lipid accumulation (Gao et al., 2019). When Erastin was applied to treat voltage-dependent anion channels (VDACs), mitochondrial function was disrupted so that oxidative substances were released, leading to oxidative death (Yagoda et al., 2007). Furthermore, Dihydroorotate Dehydrogenase (DHODH) is of great importance. In tumor cells with low GPX4 expression, DHODH activity is significantly reduced or even inactivated, which gives rise to mitochondrial lipid peroxidation accumulation and activates ferroptosis, thereby inhibiting tumor growth (Mao et al., 2021). Whereas mitochondria and glutamine come into no effect in inhibiting the GPX4-mediated pathway that boosts ferroptosis. Whether to remove the mitochondria, add electron transfer chain (ETC) inhibitors, or remove glutamine, RSL3 can inhibit GPX4-induced ferroptosis (Gaschler et al., 2018). Mitochondrial ferritin is also worth our attention. It not only inhibits oxidative stress-dependent neuronal cell damage, but also has a protective effect on Erastin-induced ferroptosis (Wang et al., 2016).

## The Mechanism of Ferroptosis

**FIGURE 1:**
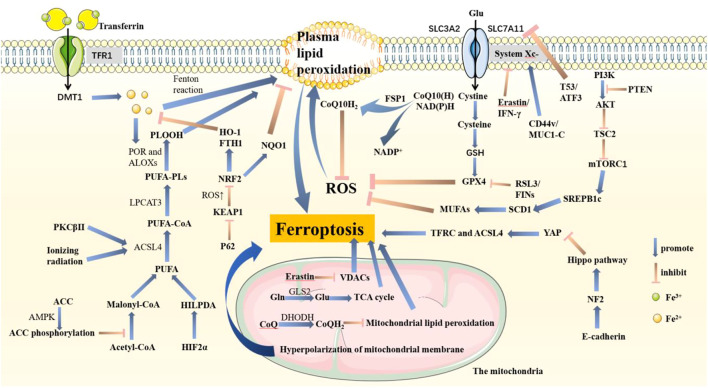
Pathways associated with ferroptosis in cells. PTEN promotes lipid peroxidation and ferroptosis by inhibiting the PI3K-AKT-mTOR pathway. System Xc^−^, composed of SLC3A2 and SLC7A11, is an important structure that helps synthesize GSH. GSH promotes GPX4 to inhibit ferroptosis. In this pathway, T53 and ATF3 acting on SLC7A11, RSL3 and FINs acting on GPX4, and Erastin and IFN-γ acting on System Xc-are important inhibitors that are conducive to ferroptosis. Also, CD44v and MUC1-C acting on System Xc-promote GPX4 synthesis. P62-KEAP1-NRF2 goes against ferroptosis by increasing NQO1, HO-1, and FTH1. Fe3^+^ binds to transferrin and enters cells through TFR1, which is reduced to Fe2^+^ and released from DMT1-mediated endosomes. Plasma lipid peroxidation is induced by the Fenton reaction process. PUFA is activated by ACSL4 and LPCAT3 and catalyzed by POR and ALOXs to promote ROS accumulation. The HIF2α-HILPDA axis, PKCβ II, and ionizing radiation both positively regulate ferroptosis, in which PKCβ II and ionizing radiation acted on ACSL4. AMPK fights ferroptosis by promoting ACC phosphorylation. E-cadherin activates the NF2-Hippo pathway and then inhibits YAP expression. Therefore, TFRC and ACSL4 are also inhibited, resulting in tumor cells growing and metastasizing more quickly. FSP1 inhibits ferroptosis by reducing CoQ10. In mitochondria, Erastin induces lipid peroxidation in both VDACs and mitochondrial potential hyperpolarization. GLS2 helps Gln transform into Glu, which enters the TCA cycle to promote ROS production. Besides, DHODH inhibits ferroptosis.

The mechanism of ferroptosis is that under the action of iron or ester oxygenase, it catalyzes the lipid peroxidation of a great deal of unsaturated fatty acids on the cell membrane. Finally, ferroptosis causes the accumulation of ROS and induces cell death (Dixon et al., 2012; Stockwell et al., 2017) (Figure 1).

### Glutathione Peroxidase (GPX4)

GPX is an indispensable peroxidase that exists widely in the body (Han et al., 2013). In the GPXs family, GPX4 takes a crucial effect on the ferroptosis regulatory pathway (Ingold et al., 2018). Its function is generally responsible for catalyzing the degradation of lipid peroxides, specifically in reducing lipid peroxides to non-toxic lipid alcohols. When the activity of GPX4 is inhibited or the amount of GPX4 is decreased, it will increase the iron-dependent reactive oxygen species in the cell, destroy the membrane structure, and induce ferroptosis (Yang et al., 2014; Seibt et al., 2019). Studies have found that GSH, in the form of reactants, participates in the process of GPX4 catalyzing the degradation of lipid peroxides (Zheng and Conrad, 2020). Accordingly, the GSH deficiency can lead to a decrease in the activity of GPX4, which in turn leads to ferroptosis. The ferroptosis inducer RL3 can also inhibit GPX4 activity by covalently binding with GPX4. The Berghe team found that through chemical proteomics experiments, RSL3 covalently bounds to the active site-containing selenocysteine ​​of GPX4. Hence RSL3 can not only directly inhibit the phospholipid peroxidase activity of GPX4, but lead to the accumulation of superoxide. At last, ferroptosis is triggered (Fang et al., 2019). It is noteworthy that GPX4 may produce unnecessary targeting effects on CD8+T cells in the anti-tumor process, leading to adverse reactions.

### Glutamate-Cystine Transporter

The glutamate-cystine transporter (system Xc-) is a heterodimer composed of SLC7A11 and SLC3A2. Cystine enters the cell via system Xc-, then GSH and GPX4 can be synthesized in the cell (Yang et al., 2014). GPX4 requires the participation of GSH in the catalytic reduction of lipid peroxides to alcohols, so inhibiting cystine uptake by cells can induce ferroptosis. Wang et al. (Wang L. et al., 2020) demonstrated that activating transcription factor 3 (ATF3) inhibits system Xc-by inhibiting the expression of SLC7A11, thereby promoting ferroptosis induced by Erastin. In addition, it is shown that pancreatic cancer cells need to take in exogenous cystine through system Xc-to prevent ferroptosis. Knockout of SLC7A11 can result in massive death of pancreatic cancer cells (Badgley et al., 2020). Lei, G. et al. revealed that ionizing radiation (IR) induces SLC7A11 and GPX4 expression as an adaptive response, which inhibits ferroptosis and enhances radiation resistance. The use of FINs, a ferroptosis inducer, restored IR sensitivity in radiation-resistant cancer cells and xenograft cells (Lei et al., 2020).

### p53

**FIGURE 2:**
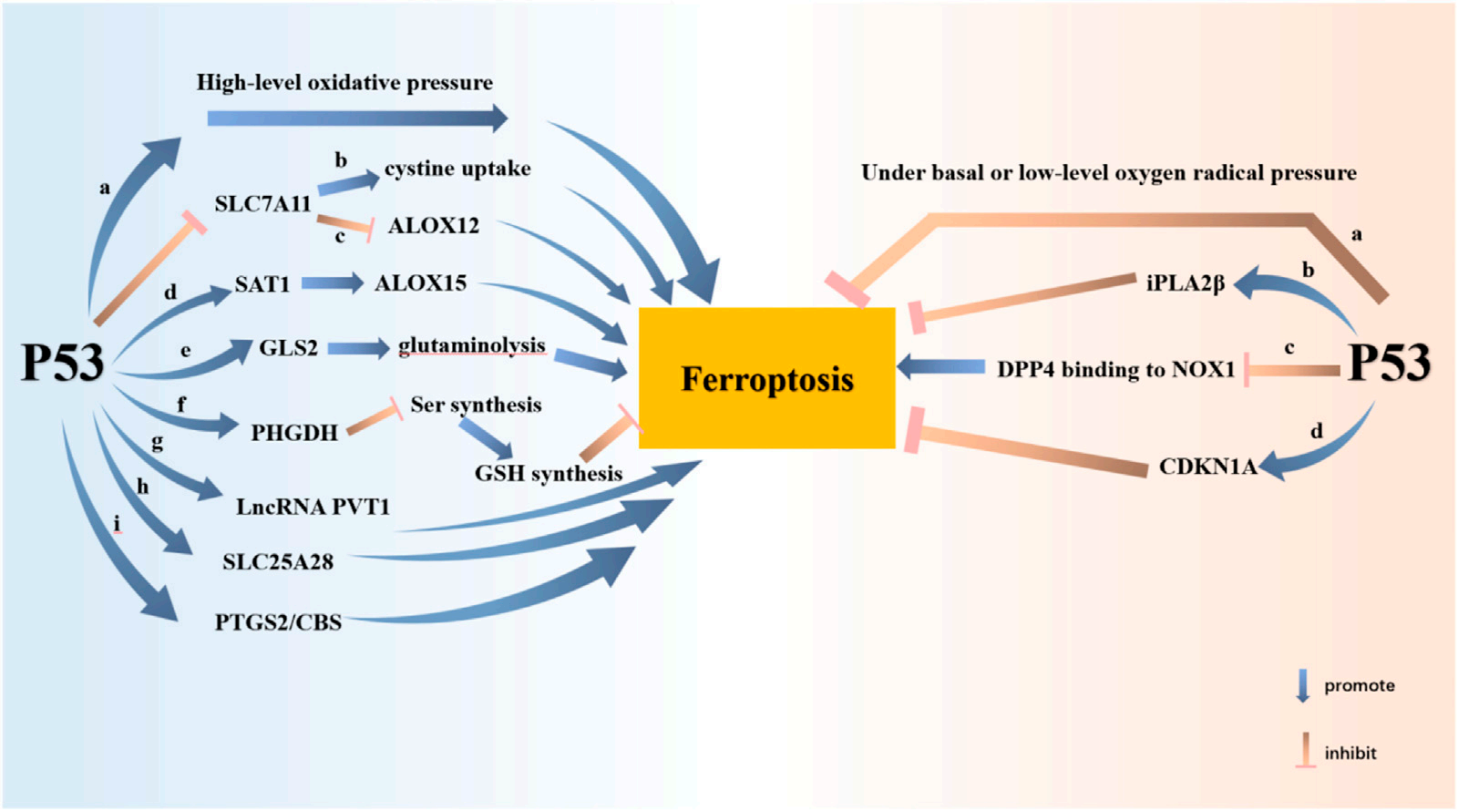
P53 potentiates ferroptosis through nine pathways and attenuates ferroptosis through three pathways. P53 promotes ferroptosis at high levels of oxidative stress. Ferroptosis is promoted by P53 inhibition of SLC7A11 expression. The consequent inhibition of cystine uptake and the release of ALOX12 contribute to ferroptosis. P53 inhibits tumor cell growth by promoting SAT1-ALOX15 and GLS-glutaminolysis axes, respectively. P53 inhibits GSH synthesis through PHGDH. P53 also promotes ferroptosis by enhancing the expression of LncRNA PVT1, SLC25A28, PTGS2 and CBS. Nevertheless, P53 inhibits ferroptosis at basal or low levels of oxygen radical pressure. P53 inhibits ferroptosis by raising the expression of iPLA2β and CDKN1A. P53 protects cells from ferroptosis by inhibiting DPP4 binding to NOX1.

p53 is an important and key tumor suppressor gene in humans, which induces cell senescence or apoptosis by regulating cell cycle arrest. Tumor suppressor activity is regulated by the classical function of p53 (Cordani et al., 2016). What’s more, p53 is capable of controlling the redox state of cells through non-classical functions. p53 regulates ferroptosis in tumor cells in a manner independent of GPX4 at high ROS levels (Chen D. et al., 2021). According to the cell environment and state, p53 has the dual effect of promoting and inhibiting ferroptosis (Hassannia et al., 2019; Sun et al., 2022; Yuan et al., 2022) (Figure 2)

1) p53 can promote ferroptosis.
  a. p53 has the effect of promoting ferroptosis under high-level oxidative pressure (Kruiswijk et al., 2015).
  b. p53 inhibits SLC7A11 transcription, then attenuates cystine uptake, and finally facilitates ferroptosis (Jiang et al., 2015).
  c. p53 acts in a GSH-independent manner. SLC7A11 is downregulated while ALOX12 is released. ALOX12 is not only a key regulator of p53-dependent ferroptosis but can be directly bound and inhibited by SLC7A11 (Chu et al., 2019).
  d. p53 can potentiate tumor ferroptosis by inducing SAT1 expression and promoting ALOX15 work (Ou et al., 2016).
  e. GLS2, the target gene of p53, catalyzes the process of Glutaminolysis to promote ferroptosis (Gao et al., 2019).
  f. p53 inhibits Ser synthesis by regulating PHGDH, thus inhibiting GSH synthesis and promoting ferroptosis.
  g. LncRNA PVT1 potentiates ferroptosis through the expression of TFR1 and p53 (Lu et al., 2020).
  h. p53 binds to the mitochondrial transporter SLC25A28 to facilitate ferroptosis (Zhang Z. et al., 2020).
  i. PTGS2 and CBS are both target genes of p53 and markers of ferroptosis.
2) p53 also negatively regulates ferroptosis in other cells or under certain conditions.
  a. p53 has the effect of inhibiting ferroptosis under basal or low-level oxygen radical pressure (Kruiswijk et al., 2015).
  b. iPLA2β mediates lipid peroxide detoxification to inhibit ROS-induced p53-driven ferroptosis in a GPX4-independent manner (Chen D. et al., 2021).
  c. In colorectal cancer, p53 directly binds DPP4 and inhibits DPP4 binding to NOX1 in the cytoplasm, resulting in inhibition of lipid peroxidation and ferroptosis in cancer cells (Xie et al., 2017).
  d. In fibrosarcoma cells, p53 induces CDKN1A expression to limit ferroptosis (Hassannia et al., 2019).

### NF2

Normal NF2 genes express ECAD, LATS1, and LATS2, which can confer resistance to ferroptosis. It is found that E-cadherin-mediated activation of intercellular NF2 and Hippo signaling pathways inhibits proto-oncogene transcription coactivator YAP. Ferroptosis is thus suppressed. This pathway is the main pathway through which cell density affects ferroptosis. If this signaling pathway is inhibited, YAP promotes cellular ferroptosis by elevating ferroptosis regulators of ACSL4 and TFRC. It is noteworthy that NF2 inhibits ferroptosis by inhibiting YAP activity, although cells expressing YAP are more sensitive to ferroptosis. As a result, malignant mutations in E-cadherin-NF2-Hippo-YAP signaling, used as biomarkers, predict the therapeutic response to ferroptosis induced in cancer (Wu et al., 2019).

The study found that silencing of NF2 activates ferroptosis-related pathways in GPX4-knockout tumor mice. NF2-silenced tumor cells grow faster without GPX4 knockout. This is because NF2 knockout facilitates tumor cell metastasis, which GPX4 knockout inhibits. These results indicated that NF2 also depended on GPX4 to regulate ferroptosis.

### FSP1 (AIFM2)

FSP1, formerly known as AIFM2, is a biomarker of ferroptosis resistance in many different cancers, protecting GPX4-deficient cells from ferroptosis. FSP1 is a key component of the non-mitochondrial coenzyme Q antioxidant system, whose inhibition of ferroptosis is mediated by ubiquinone (CoQ10) (Doll et al., 2019; Stockwell, 2019). FSP1 reduces CoQ10 to inhibit ferroptosis. Reductive CoQ is an antioxidant that traps free radicals with the ability to inhibit the propagation of lipid peroxides and prevent lipid damage. The NAD(P)H-FSP1-CoQ10 pathway is independent of the GPX4 pathway of glutathione, which synergistically inhibits phospholipid peroxidation (PLPO) and ferroptosis. Now it has been found that FSP1 is resistant to ferroptosis only when modified by cardamomylation. Cardamoylation mediates the recruitment of FSP1 into lipid droplets and the plasma membrane, where NADH-dependent CoQ is reduced (Bersuker et al., 2019). The expression of FSP1 provides a strategy for predicting the sensitivity and efficacy of cancer cells to ferroptosis-inducing chemotherapies, as well as directions for developing FSP1 inhibitors to treat cancer and overcome ferroptosis resistance in many cancers.

### AMPK

AMP-activated protein kinase (AMPK) is a crucial substance that senses and regulates the balance of cellular energy metabolism. When glucose is deficient, insufficient intracellular energy metabolism gives rise to a decrease in ATP content, which further leads to an increase in AMP/ATP ratio and activation of AMPK (Hardie et al., 2012). AMPK, which acts as the primary signaling hub to trigger energy stress, ultimately combats ferroptosis. It has been found that AMPK can block PUFAs biosynthesis regulated by Acetyl CoA carboxylase (ACC), resulting in significant inhibition of ferroptosis. As a result, on the one side, ACC phosphorylation is instrumental to regulate ferroptosis (Lee et al., 2020). On the other side, it has also been suggested that AMPK can mediate phosphorylation of Beclin 1, thereby inhibiting GSH production and promoting ferroptosis (Chen et al., 2021b).

### PI3K

The PI3K-AKT-mTOR signaling pathway, one of the most frequently mutated pathways in human cancer, prevents cancer cells from oxidative stress and ferroptosis through SREBP1/SCD1-mediated adipogenesis during carcinogenic activation (Fruman et al., 2017; Zhang et al., 2017; Zou et al., 2020). When mTORC1 activation is raised, SREBP1 can be upregulated and confer activation to SCD1 (Saxton and Sabatini, 2017). Inhibition of mTORC1 or ablation of SREBP1/SCD1 plays a protective role in ferroptosis in cancer cells with mutations in the PI3K-AKT-mTOR pathway, as well as enhances the effect of ferroptosis-induced cancer therapy. A research team discovered that drugs that block the PI3K-AKT-mTOR pathway, in combination with drugs that induce ferroptosis, evidently destroy and clear tumors (Yi J. et al., 2020).

Since both the PI3K pathway and the HDAC pathway are vital signaling pathways of malignant tumors (Li and Seto, 2016), the combination of inhibitors of the two can achieve good antitumor effects and overcome the problem of drug resistance of single drug use. A research team developed the PI3K/HDAC dual inhibitor BEBT-908, which is capable of distinctly inhibiting PI3K kinase, HDAC1, HDAC2, HDAC3, HDAC10, and HDAC11, thus delaying tumor cell growth and giving promotion cell ferroptosis. When used in association with immune checkpoint inhibitors, such as an anti-PD-1 antibody, BEBT-908 can also enhance immunotherapy efficacy and generate antitumor immune memory (Fan et al., 2021; Wang et al., 2021).

### HIF2α

Hypoxia dramatically enhances HIF2α -dependent cancer cell death and facilitates cancer cell sensitivity to ferroptosis. HIF2α inhibits GPX4 expression and drives PUFA remodeling by activating hypoxia-induced lipid droplet-associated (HILPDA), causing cancer cells to be highly sensitive to ferroptosis. As HIF2α is absent, cancer cells develop tolerance to GPX4 inhibitors and reduce the occurrence of ferroptosis. Therefore, the mechanism of cell ferroptosis driven by the HIF2α -HILPDA signaling pathway is expected to be applied in the clinic, especially in the treatment of colorectal cancer and renal clear cell carcinoma (Zou et al., 2019; Singhal et al., 2021).

## Ferroptosis and Cancer

### Overview of Ferroptosis and Tumors

Ferroptosis is a form of cell death caused by the accumulation of iron and ROS (Dixon et al., 2012). Tumors refer to neoplasms formed by the excessive proliferation of local cells under the action of various tumor-causing factors. Normally, benign tumors can be removed by surgery, and malignant tumors require radiation therapy and chemotherapy in addition to removal. Unfortunately, some tumors are highly malignant with strong drug resistance and radiation resistance. However, recent studies have found that with the deepening of research, the relationship between the proliferation of tumor cells and iron metabolism is getting closer and closer. Tumor cells cause ferroptosis through different pathways leading to their growth inhibition or death (Torti and Torti, 2020). Further investigation of ferroptosis is expected to solve the problems of cancer cell treatment resistance and drug insensitivity. Here we will systematically show the research progress of ferroptosis in cancer of different systems, providing a groundbreaking perspective for clinical treatment to inhibit and kill cancer cells.

### Ferroptosis and Respiratory Tumors

Lung cancer, the most common cancer in humans, originates in the epithelium or glands of the lungs (Berns, 2005). Mature tumor cells usually exhibit NSF1 gene dependence under circumstances of high oxygen. NSF1 maintains an iron-sulfur cluster in proteins, which are essential for tumor cells to perform basic functions. Sequentially, NSF1 protects cancer cells from oxidative damage and prevents ferroptosis. Conversely, if NSF1 and iron-sulfur clusters are inactivated, iron starvation response and ferroptosis sensitive pathways of lung cancer cells will be activated, which will induce cell ferroptosis (Nurtjahja-Tjendraputra et al., 2007; Alvarez et al., 2017). The synthesis of iron-sulfur clusters and NSF1 inhibitors may be a new method for lung cancer treatment in the future (Mao et al., 2018).

The KEAP1/NRF2 genes are highly mutated in non-small cell lung cancer (NSCLC) and tend to be found in heavy smokers. Abnormal activation of KEAP1/NRF2 can prevent cancer cells from being harmed by oxidative stress and improve the survival chance of cancer cells (Kang et al., 2021). Now Telaglenastat, a drug targeting KEAP1/NRF2 mutations, is available, offering hope for patients with NSCLC.

Furthermore, acetaminophen has been shown to induce ferroptosis by modulating the NRF2-heme oxygenase-1 signaling pathway in NSCLC (Gai et al., 2020). lncRNA P53RRA can inhibit the G1-S phase of lung cancer cells, promoting cell apoptosis and ferroptosis. By contrast, knockdown of GPX4 reverses the inhibition of ferroptosis caused by overexpression of serine/threonine/tyrosine kinase 1 and GPX4 in NSCLC (Lai et al., 2021).

### Ferroptosis and Urinary System Tumors

Ferroptosis, one of the main pathways of cell carcinoma death, has also been seen in urinary system tumors. With the interest of in-depth research on the mechanism of ferroptosis, it has the chance to be combined with targeted therapy and immunotherapy shortly, improving the success rate of urinary system tumor treatment.

Clear cell renal cell carcinoma (ccRCC) is a kind of malignant and common metastatic cancer marked by clear cytoplasm. We demonstrate that the HIF2α-HILPDA signaling pathway is the main pathway to activate ccRCC, and HIF2α strongly inhibits GPX4 expression by activating downstream proteins (Zou et al., 2019; Courtney et al., 2020; Hoefflin et al., 2020). On account of ccRCC being exceedingly sensitive to the absence of GPX4, GPX4 inhibitors can show high selective destruction (Yang and Stockwell, 2016). Not only so, the protein-modifying gene KDM5C significantly synergizes Erastin-induced lipid peroxidation and inhibits glucose to the pentose-phosphate pathway (PPP) flow and glycolysis in ccRCC. Ultimately ferroptosis is promoted while tumorigenicity is inhibited (Zheng et al., 2021). In the future, glycogen metabolism will hopefully be one of the therapeutic targets for ccRCC. Some further researches reveal that there are two new approaches for the treatment of ccRCC, which are the inhibition of glutathione synthesis and the use of fumarate hydratase, inducing ferroptosis in renal tumor cells (Kerins et al., 2018; Miess et al., 2018).

Prostate tumor is one of the most common tumors of the urinary system. For the above reason, research on the treatment of prostate cancer has been the talk of the world. Some studies have found that with the increased expression of heat shock protein (HSPB1) and ZNF217, ferroptosis of prostate cancer cells is restrained and the growth of tumor cells is synergized (Sun et al., 2015). Fe_3_O_4_ nanoparticles are surprisingly detected to trigger ferroptosis in prostate cells while avoiding damage to normal tissue. In addition, acsbg1 is a key factor regulating the transition of different modes of death, which provides new enlightenment and methods for the treatment of prostate tumors (Xie et al., 2021).

### Ferroptosis and Digestive System Tumors

The P62-KEAP1-NRF2 signaling pathway exists in hepatocellular carcinoma cells, among which NRF2 is the core transcription factor. Target genes, such as quinone oxidoreductase 1 (NQO1), heme oxygenase 1, HO-1) and ferritin heavy chain-1 (FTH1), involved in iron and ROS metabolism are up-regulated when this pathway is activated (Zhu et al., 2010; Zhu et al., 2011; Liao et al., 2013; Sun et al., 2016b). Metallothionein-1G (MT-1G) is also regulated and significantly expressed (Sun et al., 2016a). Ultimately, ferroptosis is undermined. Also, resistance to Erastin and Sorafenib in hepatocellular carcinoma is intensified. Louandre, C. et al. strongly suggested that tumor suppressor gene Rb can achieve a tumor-suppressive effect by increasing mitochondrial ROS level and ferroptosis toxicity (Louandre et al., 2015).

Pancreatic ductal adenocarcinoma (PDAC) stops ferroptosis by extracellular cysteine input despite a high concentration of lipid peroxides in cells. Cysteine outside PDAC synthesizes glutathione and CoA through the transporter SLC7A11, which counteracts the excess lipid peroxides. In contrast, when cysteine depletion occurs, intracellular autophagy promotes the degradation of the nuclear receptor Coactivator 4 (NCOA4) in PDAC. Lysosomal function and autophagy flux are impaired, thus in turn affecting cell iron overload and lipid peroxide production (Hou et al., 2016; Song et al., 2018). In other words, ferroptosis is facilitated when PDAC lacks exogenous cysteine or knocks out the cysteine transporter (SLC7A11). To our excitement, the combination of GSH and CoA, as well as metabolic disorders, that synergizes ferroptosis to treat PDAC is promising clinical option (Badgley et al., 2020). Dai, E. et al. found that a high iron diet and consumption of GPX4 can activate the TMEM173/STING-dependent DNA sensor pathway, leading to macrophages entering and activating KRAS-driven PDAC, which can significantly be inhibited by Liproxstatin -1 (Dai et al., 2020). Chen, X. et al. considered that the blockade of NUPR1, LCN2, or MGST1-mediated ALOX5 will also be a feasible strategy for the treatment of PUAC. At present, what also manifests obvious anticancer activity and promising clinical application is the combination of the antimalarial drug Artesunate and the anti-HIV1 drug Zalcitabine (Chen et al., 2021a).

In colorectal cancer, P53 not only directly inhibits DPP4 binding to NOX1 but also mediates SLC7A11, reducing the anticancer activity of Erastin *in vivo*. Whether Kras gene mutations or bromelain can stimulate ACSL4 expression, contributes to ferroptosis, and circumvents the development of colorectal cancer. With Betula etnensis Raf stimulates colorectal cancer cells, heme oxygenase (HO-1) overexpression increases intracellular iron content and redox balance was broken, followed by ferroptosis and cancer cell death (Malfa et al., 2019).

Zhang, H. et al. revealed that when processing cancer-associated fibroblasts (CAFs) of gastric cancer cells with cisplatin and paclitaxel, the USP7/hnRNPA1 axis is activated, through which miR-522 secretion is promoted. Then, the level of ferroptosis in cancer cells was downregulated in the wake of the targeted inhibition of ALOX15, and lipid peroxidation was reduced (Zhang H. et al., 2020). In other words, high levels of ALOX15 play a critical role in mediating tumor lipid peroxidation and improving overall survival in patients. Ying Liu et al. demonstrated that Jiyuan oridonin A (JDA) derivative a2 has the effect of inducing ferroptosis and anti-tumor proliferation by down-regulating GPX4 and causing iron ion accumulation. Gastric cancer drugs targeting a2 will be a hot topic in the future (Liu Y. et al., 2021). Of note, stearoyl-CoA desaturase (SCD1), perilipin2 (PLN2), and SLC7A11 can exert ferroptosis resistance in gastric cancer cells, which will be an effective target for early diagnosis, treatment, and prognosis of gastric cancer (Wang C. et al., 2020; Sun et al., 2020).

### Ferroptosis and Reproductive System Tumors

Triple-negative breast cancer (TNBC), which is famous for its high aggressiveness, high metastasis rate and high mortality, has always been the goal and direction of human efforts to explore the target of its early diagnosis and effective treatment. In TNBC, holo-lactoferrin (Holo-Lf) has been shown to inhibit MDAMB-231 cell viability while enhancing the ability of Erastin-induced cell ferroptosis. Of course, it also has an undeniable significance in improving the sensitivity of cells to radiotherapy (Zhang et al., 2021). Current studies have fully demonstrated that when MUC1-C, SREBP1, SCD1, KLF4, CD44variant, DKK1, and Cysteine are expressed in TNBC, they inhibit the occurrence of ferroptosis, resulting in high proliferation and high invasive activity (Hasegawa et al., 2016; Yi J. et al., 2020; Luis et al., 2021). MUC1-C, CD44variant, and KLF4, as negative regulators, up-regulate xCT expression and GSH level to reduce the sensitivity of tumor cells to ferroptosis (Lee et al., 2021; Zhou et al., 2021). Moreover, DKK1 regulates tumor stem cells to protect lung metastases from ferroptosis (Wu et al., 2022). Conversely, as TNBC expresses ACSL4, GPX4, DDR2, miR-324-3p, miR-3825p, and miR-5096, the imbalance of GSH levels enhances the probability of ferroptosis in cells (Lin C.-C. et al., 2021; Hou et al., 2021; Sun et al., 2021; Yadav et al., 2021). Surprisingly, the ACSL4 level can be an independent predictor of complete response and tumor-free survival after TNBC neoadjuvant chemotherapy (Dinarvand et al., 2020; Sha et al., 2021). These aforementioned proteins or genes not only have the chance to serve as novel biomarkers to predict efficacy and prognosis but are expected to become potential treatment methods for TNBC, reducing the proliferation, migration, and invasion ability of TNBC.

### Ferroptosis and Neurological System Tumors

Ferroptosis plays an incomparable key role in the characteristics and biological behavior of nervous system tumors, especially gliomas and neuroblastoma. In other words, the mechanism of ferroptosis in gliomas and neuroblastoma deserves further exploration.

One study, through analyzing 19 ferroptosis-associated genes in gliomas, found that they are closely associated with the malignancy, immunity, migration, progression, and death of gliomas. This also indicates that they are potential prognostic markers and therapeutic targets for glioma, which have a milestone significance for predicting overall survival in glioma patients and understanding the underlying mechanisms of ferroptosis (Liu et al., 2020). Chen, D. et al. manifested that in glioma, with the increase of ATF4 expression, the expression of xCT is up-regulated, resulting in three major effects. First, normal nerve cells die at an increased rate. Second, tumor cells become less sensitive to ferroptosis. Third, tumor angiogenesis and vascular structure were enhanced (Chen et al., 2017a; Chen et al., 2017b). Similarly, overexpression of NRF2 can change the tumor microenvironment, reducing the sensitivity of the tumors to ferroptosis and ferroptosis inducers and promoting tumor proliferation and migration. Concerning the glioma treatment process with Dihydroartemisinin (DHA), the PERK-ATF4 negative feedback pathway is activated. HSPA5 and GPX4 are induced to express. Most importantly, the possibility of ferroptosis of glioma cells is largely avoided. As a result, inhibition of the PERK-ATF4 pathway has a chance to significantly improve the efficiency of DHA in treating glioma (Chen et al., 2019; Yi R. et al., 2020).

Neuroblastoma, as MYC gene-driven tumors, is mainly characterized by malignancy, which is highly metastatic and prone to recurrence. Studies have shown that neuroblastoma with high expression of MYCN gene is highly dependent on cysteine and sensitive to ferroptosis. Thus, neuroblastoma can trigger ferroptosis due to cysteine deficiency and inhibition of ferroptosis by blocking cysteine uptake, transsulfuration, and inhibition of GPX4. This suggests that simultaneous targeting of multiple ferroptosis-related targets has the potential to be an effective treatment for MYCN-driven tumor therapy (Alborzinia et al., 2022).

### Ferroptosis and Melanoma

Although targeted therapy and immunotherapy have greatly improved the survival rate of melanoma patients, there are still cases of relapse and treatment failure. The discovery of ferroptosis offers hope of solving the problem. Luo, M. et al. showed that miR-137 knockdown can target SLC1A5, reducing glutamine uptake and malondialdehyde (MDA) accumulation. Finally, melanoma cells are killed by drugs due to increased sensitivity to Erastin and RSL3-induced ferroptosis (Luo et al., 2018). When melanoma cells express ACSL3, the protective effect of oleic acid on Erastin-induced ferroptosis is restored. In other words, the protection of oleic acid on tumor cells depends on ACSL3. In addition, the lymphatic environment also contributes to inhibiting ferroptosis and improves the survival of tumor cells, making it easier for tumors to metastasize distally (Ubellacker et al., 2020). Tsoi, J. et al. suggested that dedifferentiated melanoma cells are extraordinarily sensitive to ferroptosis inducers despite their obvious resistance to mitogen-activated protein kinase pathway inhibitors. Melanoma can be effectively treated with a combination of anti-melanoma drugs and ferroptosis inducers (Tsoi et al., 2018).

## Ferroptosis and Tumor Immunotherapy

Tumor immunotherapy is an incredibly innovative treatment for cancer today, which has the advantages of stronger targeting and fewer side effects (Li et al., 2020; Lin et al., 2020; Tan et al., 2020; Xu et al., 2020; Wu et al., 2021; Xu et al., 2022). Although it has incomparable advantages compared with other traditional therapies, there are still problems of poor efficacy and drug resistance (Liu X. et al., 2021; Dey et al., 2021; Repellin et al., 2021; Xiong et al., 2021). As research progresses, it is revealed that immunotherapy combined with therapies that activate ferroptosis will kill tumor cells more efficiently and quickly.

1) Zhang, H.L. et al. found that PKCβII, a sensory molecule of lipid peroxidation, promotes PUFA-phospholipid peroxide accumulation by activating ACSL4, initiating the process of inducing tumor ferroptosis. After the continuous operation of the lipid peroxide-PKCβII-ACSL4 positive feedback axis, tumor sensitivity to PD-1 antibody is significantly increased and the efficacy of immunotherapy is enhanced (Zhang et al., 2022).
2) Wang, W. et al. demonstrated that the number of CD8 (+) T cells and IFN-γ expression are positively correlated with the treatment prognosis of cancer patients. IFN-γ released by CD8 (+) T cells inhibits the expression of system Xc-, SLC3A2 and SLC7A11, thus blocking the uptake of cystine by tumor cells. This pathway enhances lipid peroxidation and promotes ferroptosis. The combination of anti-PD-L1 immunotherapy and cyst(e)kinase can effectively achieve anti-tumor immunity (Wang et al., 2019).
3) Other studies have shown that high expression and phosphorylation of TYRO3 are significantly associated with resistance to immune checkpoint inhibitors and the creation of an anti-inflammatory tumor microenvironment. TYRO3 protects tumor cells from ferroptosis through the AKT-NRF2 pathway and leads to a poor prognosis for various cancers. The combination of TYRO3-targeted drugs and anti-PD-1 drugs can not only overcome drug resistance but also reduce the therapeutic toxicity and improve the therapeutic efficacy of patients (Jiang Z. et al., 2021; Deng et al., 2022).
4) It was found that radiotherapy enhances the sensitivity of lipid peroxidation and ferroptosis by down-regulating SLC7A11 and up-regulating ACSL4 (Lang et al., 2019). In brief, radiotherapy combined with immunotherapy is conductive to treat cancer effectively (Lei et al., 2021).
5) Ma, X. et al. found that CD8 (+) T cells induce lipid peroxidation and ferroptosis mediated by CD36, resulting in reduced production of cytotoxic factors and antitumor ability of CD8 (+) T cells. Targeting CD36 restores the ability of CD8 (+) T cells to participate in tumor immunotherapy (Ma et al., 2021; Aksoylar and Patsoukis, 2022; Zhu et al., 2022).

In conclusion, the combination of immunotherapy and ferroptosis inducer has opened up a new way of thinking and strategy for cancer treatment, which will be a milestone in the improvement of cancer treatment.

## Other Novel Treatment Associated With ferroptosis

In addition to immunotherapy, many other advanced therapies conduce to induce ferroptosis and promote tumor cell development, deserving our understanding and attention. Currently, the preparation of probes and nanomedicine particles has helped to locate and observe ferroptosis and target tumor cells with precision therapy. Aniline-derived probe (Chen et al., 2018) and H-V probe (Li et al., 2019) can observe the changes of ferroptosis on cancer cells. Arginine-rich manganese silicate nanobubbles (AMSNs) (Wang et al., 2018), GPX4 covalent inhibitors (Eaton et al., 2020), SRF@FeIIITA nanoparticles (Liu et al., 2018) and hypoxia-responsive micelles (Guo et al., 2020) achieve the purpose of precise treatment of tumors. There is no doubt that, compared with the previous methods, they have their advantages and disadvantages (Table 1).

**TABLE 1:** Introduction of the more innovative methods and their respective advantages and disadvantages.

Approaches	Characteristics	Advantages	Disadvantages
Aniline-derived Probe	Lipid-derived electrophiles (LDEs) produced by Ferroptosis can influence the protein function in the manner of covalently modifying the protein. Aniline-derived probe can detect protein carbonylations and novel cysteine sites in the process of cell ferroptosis.	1. This is a commercial compound that is cheap and easy to obtain	—
		2. Compared with classic hydrazine and hydroxylamine probes, it has higher sensitivity and is very suitable for studying endogenous carbonylation modifications with weak signals	
		3. The chemical properties of the adduct of the aniline probe and the peptide are very stable, which can avoid fragmentation during sample preparation and computer application.	
Arginine-rich manganese silicate nanobubbles (AMSNs)	AMSNs is a novel tumor targeted nanoparticle that inhibits the growth of cancer cells by effectively consuming glutathione and synergistic chemotherapy drugs. During this process, the ferroptosis pathway is activated.	1. The particle size of nanobubbles is about 6.2 nm, and the potential is -17.6mv. It has a high specific surface area, porosity, colloidal stability, long half-life (4.07 h) and tumor targeting recognition function. Its lethal effect is significantly lower than that of cancer cells	1. There is still a challenge to kill cancer by consuming GSH because of the low consumption rate of GSH.
		2. AMSNs have better degradability than solid nanomaterials (such as MnO). In the process of consuming GSH, the color of AMSNs solution gradually becomes lighter, while the color of solid nanomaterials changes less, indicating that manganese ions in AMSNs are released faster and more easily degraded	—
		3. AMSNs, as a contrast agent for NMR, are easily degraded in the microenvironment of tumor cells (weak acid and high GSH concentration) and produce Mn (II) to help enhance the contrast effect of NMR T1-weighted imaging. AMSNs can be used as anti-cancer drug carriers or anti-cancer agents, effectively inhibiting the growth of cancer cells	—
Covalent inhibitor that selectively targets GPX4	The author synthesized a series of GPX4 covalent inhibitors containing electrophilic warhead and nitrile oxidation to selectively inhibit GPX4 activity and induce ferroptosis in drug-resistant tumor cells. This is a novel highly selective probe molecule for GPX4-mediated detection, providing a strategy for broadening the selection of covalent inhibitor warheads.	1. Compared with the previous covalent inhibitor-containing chloroacetamide, it has significantly superior pharmacokinetic properties	1. JKE-1674, the intermediate of ML-210, will decompose when stored in DMSO for a long time.
		2. It can more specifically induce cell ferroptosis through GPX4, and the signal pathway is single and clear. It is more suitable as a probe to study related pathways	—
Dual-function fluorescent probe (H-V)	The H-V probe can be used to detect the cytoplasmic Viscosity and OH changes during ferroptosis with a typical molecular rotor structure. With the increase of microenvironmental viscosity, the fluorescence of the probe was enhanced.	1. The unique hydroxylation of OH on aromatic compounds results in high selectivity
		2. A strong electron-donating methoxy group is added to enhance the H-V probe’s capture ability of OH, thereby improving the detection sensitivity
		3. The probe can work more effectively in the cytoplasm. The probe can detect viscosity and OH in two independent channels
		4. It has good biocompatibility
SRF@FeIIITA nanoparticles	SRF@FeIIITA nanoparticles are formed by the self-assembly of iron ions (Fe^3+^) and tannic acid (TA) on the surface of sorafenib nanocrystals. SRF inhibits GPX4 to induce ferroptosis. The Fe^2+^ sustainably reduced from TA was toxic to cancer cells. The photosensitizers assist in photodynamic therapy in conjunction with ferroptosis.	1. The prepared nanomedicine selectively causes ferroptosis of tumor cells, which is low cytotoxicity	—
		2. Many functional substances can adhere to the surface of polyphenols to facilitate the expansion of deep applications based on ferroptosis treatment methods	
Hypoxia-responsive micelles	Hypoxia-responsive micelles, acting as ferroptosis inducers, promote ferroptosis against solid tumors by reducing glutathione and thioredoxin in hypoxia.	1. Compared with other chemotherapeutic drugs (including procaspase-3 agonist, PAC-1, 1541B, nucleoside analog gemitabine, 5-F, etc.), the median lifetimewas found to be short	—
		2. The same dose of these compounds showed better than RSL3 and Erastin in inhibiting the proliferation of HCT116 and A549 cancer cells	
		3. These compounds exert their ability to inhibit tumor proliferation by inducing ferroptosis in tumor cells.	
		4. Novel structure and excellent activity.	

## Conclusion and Outlook

In summary, the discovery of ferroptosis is of epoch-making significance as it participates in the regulation of cancer in various systems through its complex mechanism (Xie et al., 2016). Researchers have developed novel therapeutic modalities based on key targets of ferroptosis pathways that complement traditional therapies and have achieved impressive results in improving treatment success, survival, and anti-tumor drug resistance in cancer patients (Chen et al., 2021b). However, ferroptosis still needs a lot of further research, especially the physiological and pathological effects, gene expression, and regulation involved in ferroptosis. First, in addition to the currently known pathway of ferroptosis, some other factors or pathways mediate ferroptosis and tumor metabolism. Second, whether there is some connection between ferroptosis and other cell death modes, such as autophagy and programmed cell death, to mediate the occurrence and development of tumors and other diseases. Third, further identification of ferroptosis-related biomarkers will contribute greatly to applying ferroptosis-based therapies to clinical cancer patients as soon as possible, as well as making precise and personalized treatment plans. Fourth, it is high time that there is a need to explore ferroptosis in association with other therapies to broaden tumor treatment regimens and slow disease progressions, such as immunotherapy, chemotherapy, and radiotherapy. Whether additional toxicity, drug resistance, and adaptation are generated during combination therapy is of public and scientific concern. The link between ferroptosis and cancer is a burgeoning area that still needs to be detected, and we still have a long way to go in the future (Wang et al., 2022).

## References

[B1] Abrams R. P. Carroll W. L. Woerpel K. A. (2016). Five-Membered Ring Peroxide Selectively Initiates Ferroptosis in Cancer Cells. ACS Chem. Biol. 11, 1305–1312. 10.1021/acschembio.5b00900 26797166 PMC5507670

[B2] Aksoylar H. I. Patsoukis N. (2022). Treatment with Exogenously Added Catalase Alters CD8 T Cell Memory Differentiation and Function. Adv. Biol. (Weinh) [Online ahead of print], e2101320. 10.1002/adbi.202101320 35481698 PMC9613814

[B3] Alborzinia H. Flórez A. F. Kreth S. Brückner L. M. Yildiz U. Gartlgruber M. (2022). MYCN Mediates Cysteine Addiction and Sensitizes Neuroblastoma to Ferroptosis. Nat. Cancer 3, 471–485. 10.1038/s43018-022-00355-4 35484422 PMC9050595

[B4] Alvarez S. W. Sviderskiy V. O. Terzi E. M. Papagiannakopoulos T. Moreira A. L. Adams S. (2017). NFS1 Undergoes Positive Selection in Lung Tumours and Protects Cells from Ferroptosis. Nature 551, 639–643. 10.1038/nature24637 29168506 PMC5808442

[B5] Anthonymuthu T. S. Kenny E. M. Shrivastava I. Tyurina Y. Y. Hier Z. E. Ting H.-C. (2018). Empowerment of 15-Lipoxygenase Catalytic Competence in Selective Oxidation of Membrane ETE-PE to Ferroptotic Death Signals, HpETE-PE. J. Am. Chem. Soc. 140, 17835–17839. 10.1021/jacs.8b09913 30525572 PMC6622169

[B6] Badgley M. A. Kremer D. M. Maurer H. C. DelGiorno K. E. Lee H.-J. Purohit V. (2020). Cysteine Depletion Induces Pancreatic Tumor Ferroptosis in Mice. Science 368, 85–89. 10.1126/science.aaw9872 32241947 PMC7681911

[B7] Berns A. (2005). Stem Cells for Lung Cancer? Cell 121, 811–813. 10.1016/j.cell.2005.06.004 15960966

[B8] Bersuker K. Hendricks J. M. Li Z. Magtanong L. Ford B. Tang P. H. (2019). The CoQ Oxidoreductase FSP1 Acts Parallel to GPX4 to Inhibit Ferroptosis. Nature 575, 688–692. 10.1038/s41586-019-1705-2 31634900 PMC6883167

[B9] Chen D. Chu B. Yang X. Liu Z. Jin Y. Kon N. (2021). iPLA2β-mediated Lipid Detoxification Controls P53-Driven Ferroptosis Independent of GPX4. Nat. Commun. 12, 3644. 10.1038/s41467-021-23902-6 34131139 PMC8206155

[B10] Chen X. Kang R. Kroemer G. Tang D. (2021a). Broadening Horizons: the Role of Ferroptosis in Cancer. Nat. Rev. Clin. Oncol. 18, 280–296. 10.1038/s41571-020-00462-0 33514910

[B11] Chen X. Kang R. Kroemer G. Tang D. (2021b). Targeting Ferroptosis in Pancreatic Cancer: a Double-Edged Sword. Trends Cancer 7, 891–901. 10.1016/j.trecan.2021.04.005 34023326

[B12] Chen D. Fan Z. Rauh M. Buchfelder M. Eyupoglu I. Y. Savaskan N. (2017a). ATF4 Promotes Angiogenesis and Neuronal Cell Death and Confers Ferroptosis in a xCT-dependent Manner. Oncogene 36, 5593–5608. 10.1038/onc.2017.146 28553953 PMC5633655

[B13] Chen D. Rauh M. Buchfelder M. Eyupoglu I. Y. Savaskan N. (2017b). The Oxido-Metabolic Driver ATF4 Enhances Temozolamide Chemo-Resistance in Human Gliomas. Oncotarget 8, 51164–51176. 10.18632/oncotarget.17737 28881638 PMC5584239

[B14] Chen Y. Liu Y. Lan T. Qin W. Zhu Y. Qin K. (2018). Quantitative Profiling of Protein Carbonylations in Ferroptosis by an Aniline-Derived Probe. J. Am. Chem. Soc. 140, 4712–4720. 10.1021/jacs.8b01462 29569437

[B15] Chen Y. Mi Y. Zhang X. Ma Q. Song Y. Zhang L. (2019). Dihydroartemisinin-induced Unfolded Protein Response Feedback Attenuates Ferroptosis via PERK/ATF4/HSPA5 Pathway in Glioma Cells. J. Exp. Clin. Cancer Res. 38, 402. 10.1186/s13046-019-1413-7 31519193 PMC6743121

[B16] Chu B. Kon N. Chen D. Li T. Liu T. Jiang L. (2019). ALOX12 Is Required for P53-Mediated Tumour Suppression through a Distinct Ferroptosis Pathway. Nat. Cell Biol. 21, 579–591. 10.1038/s41556-019-0305-6 30962574 PMC6624840

[B17] Cordani M. Oppici E. Dando I. Butturini E. Dalla Pozza E. Nadal-Serrano M. (2016). Mutant P53 Proteins Counteract Autophagic Mechanism Sensitizing Cancer Cells to mTOR Inhibition. Mol. Oncol. 10, 1008–1029. 10.1016/j.molonc.2016.04.001 27118659 PMC5423176

[B18] Courtney K. D. Ma Y. Diaz de Leon A. Christie A. Xie Z. Woolford L. (2020). HIF-2 Complex Dissociation, Target Inhibition, and Acquired Resistance with PT2385, a First-In-Class HIF-2 Inhibitor, in Patients with Clear Cell Renal Cell Carcinoma. Clin. Cancer Res. 26, 793–803. 10.1158/1078-0432.ccr-19-1459 31727677 PMC7024660

[B19] Cui W. Liu D. Gu W. Chu B. (2021). Peroxisome-driven Ether-Linked Phospholipids Biosynthesis Is Essential for Ferroptosis. Cell Death Differ. 28, 2536–2551. 10.1038/s41418-021-00769-0 33731874 PMC8329287

[B20] Dai E. Han L. Liu J. Xie Y. Zeh H. J. Kang R. (2020). Ferroptotic Damage Promotes Pancreatic Tumorigenesis through a TMEM173/STING-dependent DNA Sensor Pathway. Nat. Commun. 11, 6339. 10.1038/s41467-020-20154-8 33311482 PMC7732843

[B21] Deng J. Zhou M. Liao T. Kuang W. Xia H. Yin Z. (2022). Targeting Cancer Cell Ferroptosis to Reverse Immune Checkpoint Inhibitor Therapy Resistance. Front. Cell Dev. Biol. 10, 818453. 10.3389/fcell.2022.818453 35399527 PMC8988234

[B22] Dey M. Ayan B. Yurieva M. Unutmaz D. Ozbolat I. T. (2021). Studying Tumor Angiogenesis and Cancer Invasion in a Three-Dimensional Vascularized Breast Cancer Micro-Environment. Adv. Biol. (Weinh) 5, e2100090. 10.1002/adbi.202100090 33857356 PMC8574137

[B23] Dinarvand N. Khanahmad H. Hakimian S. M. Sheikhi A. Rashidi B. Pourfarzam M. (2020). Evaluation of Long-Chain Acyl-Coenzyme A Synthetase 4 (ACSL4) Expression in Human Breast Cancer. Res. Pharm. Sci. 15, 48–56. 10.4103/1735-5362.278714 32180816 PMC7053294

[B24] Ding H. Chen S. Pan X. Dai X. Pan G. Li Z. (2021). Transferrin Receptor 1 Ablation in Satellite Cells Impedes Skeletal Muscle Regeneration through Activation of Ferroptosis. J. Cachexia Sarcopenia Muscle 12, 746–768. 10.1002/jcsm.12700 33955709 PMC8200440

[B25] Dixon S. J. Lemberg K. M. Lamprecht M. R. Skouta R. Zaitsev E. M. Gleason C. E. (2012). Ferroptosis: an Iron-dependent Form of Nonapoptotic Cell Death. Cell 149, 1060–1072. 10.1016/j.cell.2012.03.042 22632970 PMC3367386

[B26] Doll S. Conrad M. (2017). Iron and Ferroptosis: A Still Ill‐Defined Liaison. IUBMB Life 69, 423–434. 10.1002/iub.1616 28276141

[B27] Doll S. Freitas F. P. Shah R. Aldrovandi M. da Silva M. C. Ingold I. (2019). FSP1 Is a Glutathione-independent Ferroptosis Suppressor. Nature 575, 693–698. 10.1038/s41586-019-1707-0 31634899

[B28] Eaton J. K. Furst L. Cai L. L. Viswanathan V. S. Schreiber S. L. (2020). Structure-Activity Relationships of GPX4 Inhibitor Warheads. Bioorg. Med. Chem. Lett. 30, 127538. 10.1016/j.bmcl.2020.127538 32920142 PMC8006158

[B29] Fan F. Liu P. Bao R. Chen J. Zhou M. Mo Z. (2021). A Dual PI3K/HDAC Inhibitor Induces Immunogenic Ferroptosis to Potentiate Cancer Immune Checkpoint Therapy. Cancer Res. 81, 6233–6245. 10.1158/0008-5472.can-21-1547 34711611

[B30] Fang X. Wang H. Han D. Xie E. Yang X. Wei J. (2019). Ferroptosis as a Target for Protection against Cardiomyopathy. Proc. Natl. Acad. Sci. U.S.A. 116, 2672–2680. 10.1073/pnas.1821022116 30692261 PMC6377499

[B31] Fruman D. A. Chiu H. Hopkins B. D. Bagrodia S. Cantley L. C. Abraham R. T. (2017). The PI3K Pathway in Human Disease. Cell 170, 605–635. 10.1016/j.cell.2017.07.029 28802037 PMC5726441

[B32] Gai C. Yu M. Li Z. Wang Y. Ding D. Zheng J. (2020). Acetaminophen Sensitizing Erastin‐Induced Ferroptosis via Modulation of Nrf2/Heme Oxygenase‐1 Signaling Pathway in Non‐Small‐Cell Lung Cancer. J. Cell Physiol. 235, 3329–3339. 10.1002/jcp.29221 31541463 PMC13482668

[B33] Gao M. Yi J. Zhu J. Minikes A. M. Monian P. Thompson C. B. (2019). Role of Mitochondria in Ferroptosis. Mol. Cell 73, 354–363. 10.1016/j.molcel.2018.10.042 30581146 PMC6338496

[B34] Gaschler M. M. Hu F. Feng H. Linkermann A. Min W. Stockwell B. R. (2018). Determination of the Subcellular Localization and Mechanism of Action of Ferrostatins in Suppressing Ferroptosis. ACS Chem. Biol. 13, 1013–1020. 10.1021/acschembio.8b00199 29512999 PMC5960802

[B35] Guo X. Liu F. Deng J. Dai P. Qin Y. Li Z. (2020). Electron-Accepting Micelles Deplete Reduced Nicotinamide Adenine Dinucleotide Phosphate and Impair Two Antioxidant Cascades for Ferroptosis-Induced Tumor Eradication. ACS Nano 14, 14715–14730. 10.1021/acsnano.0c00764 33156626

[B36] Han X. Fan Z. Yu Y. Liu S. Hao Y. Huo R. (2013). Expression and Characterization of Recombinant Human Phospholipid Hydroperoxide Glutathione Peroxidase. IUBMB Life 65, 951–956. 10.1002/iub.1220 24170573

[B37] Hardie D. G. Ross F. A. Hawley S. A. (2012). AMPK: a Nutrient and Energy Sensor that Maintains Energy Homeostasis. Nat. Rev. Mol. Cell Biol. 13, 251–262. 10.1038/nrm3311 22436748 PMC5726489

[B38] Hasegawa M. Takahashi H. Rajabi H. Alam M. Suzuki Y. Yin L. (2016). Functional Interactions of the Cystine/Glutamate Antiporter, CD44v and MUC1-C Oncoprotein in Triple-Negative Breast Cancer Cells. Oncotarget 7, 11756–11769. 10.18632/oncotarget.7598 26930718 PMC4914246

[B39] Hassannia B. Vandenabeele P. Vanden Berghe T. (2019). Targeting Ferroptosis to Iron Out Cancer. Cancer Cell 35, 830–849. 10.1016/j.ccell.2019.04.002 31105042

[B40] Hoefflin R. Harlander S. Schäfer S. Metzger P. Kuo F. Schönenberger D. (2020). HIF-1α and HIF-2α Differently Regulate Tumour Development and Inflammation of Clear Cell Renal Cell Carcinoma in Mice. Nat. Commun. 11, 4111. 10.1038/s41467-020-17873-3 32807776 PMC7431415

[B41] Hou W. Xie Y. Song X. Sun X. Lotze M. T. Zeh H. J. 3rd (2016). Autophagy Promotes Ferroptosis by Degradation of Ferritin. Autophagy 12, 1425–1428. 10.1080/15548627.2016.1187366 27245739 PMC4968231

[B42] Hou Y. Cai S. Yu S. Lin H. (2021). Metformin Induces Ferroptosis by Targeting miR-324-3p/GPX4 axis in Breast Cancer. Acta Biochim. Biophys. Sin. (Shanghai) 53, 333–341. 10.1093/abbs/gmaa180 33522578

[B43] Hwang J. S. Kim E. Lee H. G. Lee W. J. Won J. P. Hur J. (2021). Peroxisome Proliferator-Activated Receptor δ Rescues xCT-Deficient Cells from Ferroptosis by Targeting Peroxisomes. Biomed. Pharmacother. 143, 112223. 10.1016/j.biopha.2021.112223 34649350

[B44] Ingold I. Berndt C. Schmitt S. Doll S. Poschmann G. Buday K. (2018). Selenium Utilization by GPX4 Is Required to Prevent Hydroperoxide-Induced Ferroptosis. Cell 172, 409–422. 10.1016/j.cell.2017.11.048 29290465

[B45] Jiang L. Kon N. Li T. Wang S.-J. Su T. Hibshoosh H. (2015). Ferroptosis as a P53-Mediated Activity during Tumour Suppression. Nature 520, 57–62. 10.1038/nature14344 25799988 PMC4455927

[B46] Jiang X. Stockwell B. R. Conrad M. (2021). Ferroptosis: Mechanisms, Biology and Role in Disease. Nat. Rev. Mol. Cell Biol. 22, 266–282. 10.1038/s41580-020-00324-8 33495651 PMC8142022

[B47] Jiang Z. Lim S. O. Yan M. Hsu J. L. Yao J. Wei Y. (2021). TYRO3 Induces Anti-PD-1/pd-L1 Therapy Resistance by Limiting Innate Immunity and Tumoral Ferroptosis. J. Clin. Invest 131, e139434. 10.1172/jci139434 PMC8262501 33855973

[B48] Kang Y. P. Mockabee-Macias A. Jiang C. Falzone A. Prieto-Farigua N. Stone E. (2021). Non-canonical Glutamate-Cysteine Ligase Activity Protects against Ferroptosis. Cell Metab. 33, 174–189. 10.1016/j.cmet.2020.12.007 33357455 PMC7839835

[B49] Kerins M. J. Milligan J. Wohlschlegel J. A. Ooi A. (2018). Fumarate Hydratase Inactivation in Hereditary Leiomyomatosis and Renal Cell Cancer Is Synthetic Lethal with Ferroptosis Induction. Cancer Sci. 109, 2757–2766. 10.1111/cas.13701 29917289 PMC6125459

[B50] Kruiswijk F. Labuschagne C. F. Vousden K. H. (2015). p53 in Survival, Death and Metabolic Health: a Lifeguard with a Licence to Kill. Nat. Rev. Mol. Cell Biol. 16, 393–405. 10.1038/nrm4007 26122615

[B51] Lai Y. Lin F. Wang X. Zhang J. Xia J. Sun Y. (2021). STYK1/NOK Promotes Metastasis and Epithelial-Mesenchymal Transition in Non-small Cell Lung Cancer by Suppressing FoxO1 Signaling. Front. Cell Dev. Biol. 9, 621147. 10.3389/fcell.2021.621147 34295886 PMC8290174

[B52] Lang X. Green M. D. Wang W. Yu J. Choi J. E. Jiang L. (2019). Radiotherapy and Immunotherapy Promote Tumoral Lipid Oxidation and Ferroptosis via Synergistic Repression of SLC7A11. Cancer Discov. 9, 1673–1685. 10.1158/2159-8290.cd-19-0338 31554642 PMC6891128

[B53] Lee H. Zandkarimi F. Zhang Y. Meena J. K. Kim J. Zhuang L. (2020). Energy-Stress-Mediated AMPK Activation Inhibits Ferroptosis. Nat. Cell Biol. 22, 225–234. 10.1038/s41556-020-0461-8 32029897 PMC7008777

[B54] Lee N. Carlisle A. E. Peppers A. Park S. J. Doshi M. B. Spears M. E. (2021). xCT-Driven Expression of GPX4 Determines Sensitivity of Breast Cancer Cells to Ferroptosis Inducers. Antioxidants (Basel) 10, 317. 10.3390/antiox10020317 33672555 PMC7923775

[B55] Lei G. Mao C. Yan Y. Zhuang L. Gan B. (2021). Ferroptosis, Radiotherapy, and Combination Therapeutic Strategies. Protein Cell 12, 836–857. 10.1007/s13238-021-00841-y 33891303 PMC8563889

[B56] Lei G. Zhang Y. Koppula P. Liu X. Zhang J. Lin S. H. (2020). The Role of Ferroptosis in Ionizing Radiation-Induced Cell Death and Tumor Suppression. Cell Res. 30, 146–162. 10.1038/s41422-019-0263-3 31949285 PMC7015061

[B57] Li H. Shi W. Li X. Hu Y. Fang Y. Ma H. (2019). Ferroptosis Accompanied by •OH Generation and Cytoplasmic Viscosity Increase Revealed via Dual-Functional Fluorescence Probe. J. Am. Chem. Soc. 141, 18301–18307. 10.1021/jacs.9b09722 31644876

[B58] Li S. Zhang Z. Lai W.-F. Cui L. Zhu X. (2020). How to Overcome the Side Effects of Tumor Immunotherapy. Biomed. Pharmacother. 130, 110639. 10.1016/j.biopha.2020.110639 33658124

[B59] Li Y. Seto E. (2016). HDACs and HDAC Inhibitors in Cancer Development and Therapy. Cold Spring Harb. Perspect. Med. 6, a026831. 10.1101/cshperspect.a026831 27599530 PMC5046688

[B60] Liao Y.-F. Zhu W. Li D. P. Zhu X. (2013). Heme Oxygenase-1 and Gut Ischemia/Reperfusion Injury: A Short Review.World J Gastroenterol. 19, 3555–3561. 10.3748/wjg.v19.i23.3555 23801856 PMC3691047

[B61] Lin B. Du L. Li H. Zhu X. Cui L. Li X. (2020). Tumor-infiltrating Lymphocytes: Warriors Fight against Tumors Powerfully. Biomed. Pharmacother. 132, 110873. 10.1016/j.biopha.2020.110873 33068926

[B62] Lin C.-C. Yang W.-H. Lin Y.-T. Tang X. Chen P.-H. Ding C.-K. C. (2021. DDR2 Upregulation Confers Ferroptosis Susceptibility of Recurrent Breast Tumors through the Hippo Pathway. Oncogene 40, 2018–2034. 10.1038/s41388-021-01676-x 33603168 PMC7988308

[B63] Lin Z. Liu J. Kang R. Yang M. Tang D. (2021). Lipid Metabolism in Ferroptosis. Adv. Biol. (Weinh) 5, e2100396. 10.1002/adbi.202100396 34015188

[B64] Liu H.-j. Hu H.-m. Li G.-z. Zhang Y. Wu F. Liu X. (2020). Ferroptosis-Related Gene Signature Predicts Glioma Cell Death and Glioma Patient Progression. Front. Cell Dev. Biol. 8, 538. 10.3389/fcell.2020.00538 32733879 PMC7363771

[B65] Liu T. Liu W. Zhang M. Yu W. Gao F. Li C. (2018). Ferrous-Supply-Regeneration Nanoengineering for Cancer-cell-specific Ferroptosis in Combination with Imaging-Guided Photodynamic Therapy. ACS Nano 12, 12181–12192. 10.1021/acsnano.8b05860 30458111

[B66] Liu X. Li Z. Wang Y. (2021). Advances in Targeted Therapy and Immunotherapy for Pancreatic Cancer. Adv. Biol. (Weinh) 5, e1900236. 10.1002/adbi.201900236 33729700

[B67] Liu Y. Song Z. Liu Y. Ma X. Wang W. Ke Y. (2021). Identification of Ferroptosis as a Novel Mechanism for Antitumor Activity of Natural Product Derivative A2 in Gastric Cancer. Acta Pharm. Sin. B 11, 1513–1525. 10.1016/j.apsb.2021.05.006 34221865 PMC8245858

[B68] Louandre C. Marcq I. Bouhlal H. Lachaier E. Godin C. Saidak Z. (2015). The Retinoblastoma (Rb) Protein Regulates Ferroptosis Induced by Sorafenib in Human Hepatocellular Carcinoma Cells. Cancer Lett. 356, 971–977. 10.1016/j.canlet.2014.11.014 25444922

[B69] Lu J. Xu F. Lu H. (2020). LncRNA PVT1 Regulates Ferroptosis through miR-214-Mediated TFR1 and P53. Life Sci. 260, 118305. 10.1016/j.lfs.2020.118305 32827544

[B70] Luis G. Godfroid A. Nishiumi S. Cimino J. Blacher S. Maquoi E. (2021). Tumor Resistance to Ferroptosis Driven by Stearoyl-CoA Desaturase-1 (SCD1) in Cancer Cells and Fatty Acid Biding Protein-4 (FABP4) in Tumor Microenvironment Promote Tumor Recurrence. Redox Biol. 43, 102006. 10.1016/j.redox.2021.102006 34030117 PMC8163990

[B71] Luo M. Wu L. Zhang K. Wang H. Zhang T. Gutierrez L. (2018). miR-137 Regulates Ferroptosis by Targeting Glutamine Transporter SLC1A5 in Melanoma. Cell Death Differ. 25, 1457–1472. 10.1038/s41418-017-0053-8 29348676 PMC6113319

[B72] Ma X. Xiao L. Liu L. Ye L. Su P. Bi E. (2021). CD36-mediated Ferroptosis Dampens Intratumoral CD8^+^ T Cell Effector Function and Impairs Their Antitumor Ability. Cell Metab. 33, 1001–1012. 10.1016/j.cmet.2021.02.015 33691090 PMC8102368

[B73] Malfa G. A. Tomasello B. Acquaviva R. Genovese C. La Mantia A. Cammarata F. P. (2019). Betula Etnensis Raf. (Betulaceae) Extract Induced HO-1 Expression and Ferroptosis Cell Death in Human Colon Cancer Cells. Int. J. Mol. Sci. 20, 2723. 10.3390/ijms20112723 PMC6600233 31163602

[B74] Mao C. Liu X. Zhang Y. Lei G. Yan Y. Lee H. (2021). DHODH-Mediated Ferroptosis Defence Is a Targetable Vulnerability in Cancer. Nature 593, 586–590. 10.1038/s41586-021-03539-7 33981038 PMC8895686

[B75] Mao C. Wang X. Liu Y. Wang M. Yan B. Jiang Y. (2018). A G3BP1-Interacting lncRNA Promotes Ferroptosis and Apoptosis in Cancer via Nuclear Sequestration of P53. Cancer Res. 78, 3484–3496. 10.1158/0008-5472.can-17-3454 29588351 PMC8073197

[B76] Miess H. Dankworth B. Gouw A. M. Rosenfeldt M. Schmitz W. Jiang M. (2018). The Glutathione Redox System Is Essential to Prevent Ferroptosis Caused by Impaired Lipid Metabolism in Clear Cell Renal Cell Carcinoma. Oncogene 37, 5435–5450. 10.1038/s41388-018-0315-z 29872221 PMC6173300

[B77] Nieto-Garai J. A. Contreras F. X. Arboleya A. Lorizate M. (2022). Role of Protein-Lipid Interactions in Viral Entry. Adv. Biol. (Weinh) 6, e2101264. 10.1002/adbi.202101264 35119227

[B78] Nurtjahja-Tjendraputra E. Fu D. Phang J. M. Richardson D. R. (2007). Iron Chelation Regulates Cyclin D1 Expression via the Proteasome: a Link to Iron Deficiency-Mediated Growth Suppression. Blood 109, 4045–4054. 10.1182/blood-2006-10-047753 17197429

[B79] Ou Y. Wang S. J. Li D. Chu B. Gu W. (2016). Activation of SAT1 Engages Polyamine Metabolism with P53-Mediated Ferroptotic Responses. Proc. Natl. Acad. Sci. U. S. A. 113, E6806–E6812. 10.1073/pnas.1607152113 27698118 PMC5098629

[B80] Repellin C. E. Ssemadaali M. A. Newmyer S. Radhakrishnan H. Javitz H. S. Bhatnagar P. (2021). NK-cell Biofactory as an Off-The-Shelf Cell-Based Vector for Targeted *In Situ* Synthesis of Engineered Proteins. Adv. Biol. (Weinh) 5, e2000298. 10.1002/adbi.202000298 33871182 PMC8275051

[B81] Ruiz-de-Angulo A. Bilbao-Asensio M. Cronin J. Evans S. J. Clift M. J. D. Llop J. (2020). Chemically Programmed Vaccines: Iron Catalysis in Nanoparticles Enhances Combination Immunotherapy and Immunotherapy-Promoted Tumor Ferroptosis. iScience 23, 101499. 10.1016/j.isci.2020.101499 32919370 PMC7490994

[B82] Saxton R. A. Sabatini D. M. (2017). mTOR Signaling in Growth, Metabolism, and Disease. Cell 169, 361–371. 10.1016/j.cell.2017.03.035 28388417

[B83] Seibt T. M. Proneth B. Conrad M. (2019). Role of GPX4 in Ferroptosis and its Pharmacological Implication. Free Radic. Biol. Med. 133, 144–152. 10.1016/j.freeradbiomed.2018.09.014 30219704

[B84] Sha R. Xu Y. Yuan C. Sheng X. Wu Z. Peng J. (2021). Predictive and Prognostic Impact of Ferroptosis-Related Genes ACSL4 and GPX4 on Breast Cancer Treated with Neoadjuvant Chemotherapy. EBioMedicine 71, 103560. 10.1016/j.ebiom.2021.103560 34482070 PMC8417304

[B85] Singhal R. Mitta S. R. Das N. K. Kerk S. A. Sajjakulnukit P. Solanki S. (2021). HIF-2α Activation Potentiates Oxidative Cell Death in Colorectal Cancers by Increasing Cellular Iron. J. Clin. Invest 131, e143691. 10.1172/JCI143691 PMC8203462 33914705

[B86] Song X. Zhu S. Chen P. Hou W. Wen Q. Liu J. (2018). AMPK-Mediated BECN1 Phosphorylation Promotes Ferroptosis by Directly Blocking System X_c_ ^-^ Activity. Curr. Biol. 28, 2388–2399. e2385. 10.1016/j.cub.2018.05.094 30057310 PMC6081251

[B87] Stockwell B. R. (2019). A Powerful Cell-Protection System Prevents Cell Death by Ferroptosis. Nature 575, 597–598. 10.1038/d41586-019-03145-8 31768036 PMC7262969

[B88] Stockwell B. R. Friedmann Angeli J. P. Bayir H. Bush A. I. Conrad M. Dixon S. J. (2017). Ferroptosis: A Regulated Cell Death Nexus Linking Metabolism, Redox Biology, and Disease. Cell 171, 273–285. 10.1016/j.cell.2017.09.021 28985560 PMC5685180

[B89] Sun D. Li Y.-C. Zhang X.-Y. (2021). Lidocaine Promoted Ferroptosis by Targeting miR-382-5p/SLC7A11 Axis in Ovarian and Breast Cancer. Front. Pharmacol. 12, 681223. 10.3389/fphar.2021.681223 34122108 PMC8188239

[B90] Sun Q. Xu Y. Yuan F. e. Qi Y. Wang Y. Chen Q. (2022). Rho Family GTPase 1 (RND1), a Novel Regulator of P53, Enhances Ferroptosis in Glioblastoma. Cell Biosci. 12, 53. 10.1186/s13578-022-00791-w 35505371 PMC9066768

[B91] Sun X. Niu X. Chen R. He W. Chen D. Kang R. (2016a). Metallothionein‐1G Facilitates Sorafenib Resistance through Inhibition of Ferroptosis. Hepatology 64, 488–500. 10.1002/hep.28574 27015352 PMC4956496

[B92] Sun X. Ou Z. Chen R. Niu X. Chen D. Kang R. (2016b). Activation of the P62-Keap1-NRF2 Pathway Protects against Ferroptosis in Hepatocellular Carcinoma Cells. Hepatology 63, 173–184. 10.1002/hep.28251 26403645 PMC4688087

[B93] Sun X. Ou Z. Xie M. Kang R. Fan Y. Niu X. (2015). HSPB1 as a Novel Regulator of Ferroptotic Cancer Cell Death. Oncogene 34, 5617–5625. 10.1038/onc.2015.32 25728673 PMC4640181

[B94] Sun X. Yang S. Feng X. Zheng Y. Zhou J. Wang H. (2020). The Modification of Ferroptosis and Abnormal Lipometabolism through Overexpression and Knockdown of Potential Prognostic Biomarker Perilipin2 in Gastric Carcinoma. Gastric Cancer 23, 241–259. 10.1007/s10120-019-01004-z 31520166

[B95] Tan S. Li D. Zhu X. (2020). Cancer Immunotherapy: Pros, Cons and beyond. Biomed. Pharmacother. 124, 109821. 10.1016/j.biopha.2020.109821 31962285

[B96] Torti S. V. Torti F. M. (2020). Iron: The Cancer Connection. Mol. Aspects Med. 75, 100860. 10.1016/j.mam.2020.100860 32340745 PMC9107937

[B97] Tsoi J. Robert L. Paraiso K. Galvan C. Sheu K. M. Lay J. (2018). Multi-stage Differentiation Defines Melanoma Subtypes with Differential Vulnerability to Drug-Induced Iron-Dependent Oxidative Stress. Cancer Cell 33, 890–904. 10.1016/j.ccell.2018.03.017 29657129 PMC5953834

[B98] Ubellacker J. M. Tasdogan A. Ramesh V. Shen B. Mitchell E. C. Martin-Sandoval M. S. (2020). Lymph Protects Metastasizing Melanoma Cells from Ferroptosis. Nature 585, 113–118. 10.1038/s41586-020-2623-z 32814895 PMC7484468

[B99] Venkatesh D. O'Brien N. A. Zandkarimi F. Tong D. R. Stokes M. E. Dunn D. E. (2020). MDM2 and MDMX Promote Ferroptosis by PPARα-Mediated Lipid Remodeling. Genes Dev. 34, 526–543. 10.1101/gad.334219.119 32079652 PMC7111265

[B100] Wang C. Shi M. Ji J. Cai Q. Zhao Q. Jiang J. (2020). Stearoyl-CoA Desaturase 1 (SCD1) Facilitates the Growth and Anti-ferroptosis of Gastric Cancer Cells and Predicts Poor Prognosis of Gastric Cancer. Aging 12, 15374–15391. 10.18632/aging.103598 32726752 PMC7467382

[B101] Wang L. Liu Y. Du T. Yang H. Lei L. Guo M. (2020). ATF3 Promotes Erastin-Induced Ferroptosis by Suppressing System Xc-. Cell Death Differ. 27, 662–675. 10.1038/s41418-019-0380-z 31273299 PMC7206049

[B102] Wang H.-T. Ju J. Wang S.-C. Zhang Y.-H. Liu C.-Y. Wang T. (2022). Insights into Ferroptosis, a Novel Target for the Therapy of Cancer. Front. Oncol. 12, 812534. 10.3389/fonc.2022.812534 35280796 PMC8914339

[B103] Wang Q. Bardhan K. Boussiotis V. A. Patsoukis N. (2021). The PD-1 Interactome. Adv. Biol. (Weinh) 5, e2100758. 10.1002/adbi.202100758 34170628 PMC10754315

[B104] Wang S. Li F. Qiao R. Hu X. Liao H. Chen L. (2018). Arginine-Rich Manganese Silicate Nanobubbles as a Ferroptosis-Inducing Agent for Tumor-Targeted Theranostics. ACS Nano 12, 12380–12392. 10.1021/acsnano.8b06399 30495919

[B105] Wang W. Green M. Choi J. E. Gijón M. Kennedy P. D. Johnson J. K. (2019). CD8+ T Cells Regulate Tumour Ferroptosis during Cancer Immunotherapy. Nature 569, 270–274. 10.1038/s41586-019-1170-y 31043744 PMC6533917

[B106] Wang Y.-Q. Chang S.-Y. Wu Q. Gou Y.-J. Jia L. Cui Y.-M. (2016). The Protective Role of Mitochondrial Ferritin on Erastin-Induced Ferroptosis. Front. Aging Neurosci. 8, 308. 10.3389/fnagi.2016.00308 28066232 PMC5167726

[B107] Wei C. Li M. Li X. Lyu J. Zhu X. (2022). Phase Separation: "The Master Key" to Deciphering the Physiological and Pathological Functions of Cells. Adv. Biol. (Weinh), e2200006. 10.1002/adbi.202200006 35514065

[B108] Wu J. Minikes A. M. Gao M. Bian H. Li Y. Stockwell B. R. (2019). Intercellular Interaction Dictates Cancer Cell Ferroptosis via NF2-YAP Signalling. Nature 572, 402–406. 10.1038/s41586-019-1426-6 31341276 PMC6697195

[B109] Wu M. Zhang X. Zhang W. Chiou Y. S. Qian W. Liu X. (2022). Cancer Stem Cell Regulated Phenotypic Plasticity Protects Metastasized Cancer Cells from Ferroptosis. Nat. Commun. 13, 1371. 10.1038/s41467-022-29018-9 35296660 PMC8927306

[B110] Wu Z. Li S. Zhu X. (2021). The Mechanism of Stimulating and Mobilizing the Immune System Enhancing the Anti-tumor Immunity. Front. Immunol. 12, 682435. 10.3389/fimmu.2021.682435 34194437 PMC8237941

[B111] Xie S. Sun W. Zhang C. Dong B. Yang J. Hou M. (2021). Metabolic Control by Heat Stress Determining Cell Fate to Ferroptosis for Effective Cancer Therapy. ACS Nano 15, 7179–7194. 10.1021/acsnano.1c00380 33861924

[B112] Xie Y. Hou W. Song X. Yu Y. Huang J. Sun X. (2016). Ferroptosis: Process and Function. Cell Death Differ. 23, 369–379. 10.1038/cdd.2015.158 26794443 PMC5072448

[B113] Xie Y. Zhu S. Song X. Sun X. Fan Y. Liu J. (2017). The Tumor Suppressor P53 Limits Ferroptosis by Blocking DPP4 Activity. Cell Rep. 20, 1692–1704. 10.1016/j.celrep.2017.07.055 28813679

[B114] Xiong J. Wang H. Wang Q. (2021). Suppressive Myeloid Cells Shape the Tumor Immune Microenvironment. Adv. Biol. (Weinh) 5, e1900311. 10.1002/adbi.201900311 33729699

[B115] Xu J. Li X. Du Y. (2022). Antibody-Pattern Recognition Receptor Agonist Conjugates: A Promising Therapeutic Strategy for Cancer. Adv. Biol. (Weinh) 6, e2101065. 10.1002/adbi.202101065 35122418

[B116] Xu P. Luo H. Kong Y. Lai W.-F. Cui L. Zhu X. (2020). Cancer Neoantigen: Boosting Immunotherapy. Biomed. Pharmacother. 131, 110640. 10.1016/j.biopha.2020.110640 32836075

[B117] Yadav P. Sharma P. Sundaram S. Venkatraman G. Bera A. K. Karunagaran D. (2021). SLC7A11/xCT Is a Target of miR-5096 and its Restoration Partially Rescues miR-5096-Mediated Ferroptosis and Anti-tumor Effects in Human Breast Cancer Cells. Cancer Lett. 522, 211–224. 10.1016/j.canlet.2021.09.033 34571083

[B118] Yagoda N. von Rechenberg M. Zaganjor E. Bauer A. J. Yang W. S. Fridman D. J. (2007). RAS-RAF-MEK-dependent Oxidative Cell Death Involving Voltage-dependent Anion Channels. Nature 447, 864–868. 10.1038/nature05859 17568748 PMC3047570

[B119] Yan B. Ai Y. Sun Q. Ma Y. Cao Y. Wang J. (2021). Membrane Damage during Ferroptosis Is Caused by Oxidation of Phospholipids Catalyzed by the Oxidoreductases POR and CYB5R1. Mol. Cell 81, 355–369. 10.1016/j.molcel.2020.11.024 33321093

[B120] Yang W. S. Kim K. J. Gaschler M. M. Patel M. Shchepinov M. S. Stockwell B. R. (2016). Peroxidation of Polyunsaturated Fatty Acids by Lipoxygenases Drives Ferroptosis. Proc. Natl. Acad. Sci. U. S. A. 113, E4966–E4975. 10.1073/pnas.1603244113 27506793 PMC5003261

[B121] Yang W. S. SriRamaratnam R. Welsch M. E. Shimada K. Skouta R. Viswanathan V. S. (2014). Regulation of Ferroptotic Cancer Cell Death by GPX4. Cell 156, 317–331. 10.1016/j.cell.2013.12.010 24439385 PMC4076414

[B122] Yang W. S. Stockwell B. R. (2016). Ferroptosis: Death by Lipid Peroxidation. Trends Cell Biol. 26, 165–176. 10.1016/j.tcb.2015.10.014 26653790 PMC4764384

[B123] Yi J. Zhu J. Wu J. Thompson C. B. Jiang X. (2020). Oncogenic Activation of PI3K-AKT-mTOR Signaling Suppresses Ferroptosis via SREBP-Mediated Lipogenesis. Proc. Natl. Acad. Sci. U.S.A. 117, 31189–31197. 10.1073/pnas.2017152117 33229547 PMC7733797

[B124] Yi R. Wang H. Deng C. Wang X. Yao L. Niu W. (2020). Dihydroartemisinin Initiates Ferroptosis in Glioblastoma through GPX4 Inhibition. Biosci. Rep. 40. 10.1042/BSR20193314 PMC7313443 32452511

[B125] Yuan F. Sun Q. Zhang S. Ye L. Xu Y. Deng G. (2022). The Dual Role of P62 in Ferroptosis of Glioblastoma According to P53 Status. Cell Biosci. 12, 20. 10.1186/s13578-022-00764-z 35216629 PMC8881833

[B126] Zhang H.-L. Hu B.-X. Li Z.-L. Du T. Shan J.-L. Ye Z.-P. (2022). PKCβII Phosphorylates ACSL4 to Amplify Lipid Peroxidation to Induce Ferroptosis. Nat. Cell Biol. 24, 88–98. 10.1038/s41556-021-00818-3 35027735

[B127] Zhang H. Deng T. Liu R. Ning T. Yang H. Liu D. (2020). CAF Secreted miR-522 Suppresses Ferroptosis and Promotes Acquired Chemo-Resistance in Gastric Cancer. Mol. Cancer 19, 43. 10.1186/s12943-020-01168-8 32106859 PMC7045485

[B128] Zhang Z. Guo M. Shen M. Kong D. Zhang F. Shao J. (2020). The BRD7-P53-Slc25a28 axis Regulates Ferroptosis in Hepatic Stellate Cells. Redox Biol. 36, 101619. 10.1016/j.redox.2020.101619 32863216 PMC7330619

[B129] Zhang Y. Kwok-Shing Ng P. Kucherlapati M. Chen F. Liu Y. Tsang Y. H. (2017). A Pan-Cancer Proteogenomic Atlas of PI3K/AKT/mTOR Pathway Alterations. Cancer Cell 31, 820–832. e823. 10.1016/j.ccell.2017.04.013 28528867 PMC5502825

[B130] Zhang Z. Lu M. Chen C. Tong X. Li Y. Yang K. (2021). Holo-lactoferrin: the Link between Ferroptosis and Radiotherapy in Triple-Negative Breast Cancer. Theranostics 11, 3167–3182. 10.7150/thno.52028 33537080 PMC7847686

[B131] Zheng J. Conrad M. (2020). The Metabolic Underpinnings of Ferroptosis. Cell Metab. 32, 920–937. 10.1016/j.cmet.2020.10.011 33217331

[B132] Zheng Q. Li P. Zhou X. Qiang Y. Fan J. Lin Y. (2021). Deficiency of the X-Inactivation Escaping Gene KDM5C in Clear Cell Renal Cell Carcinoma Promotes Tumorigenicity by Reprogramming Glycogen Metabolism and Inhibiting Ferroptosis. Theranostics 11, 8674–8691. 10.7150/thno.60233 34522206 PMC8419058

[B133] Zhou Y. Yang J. Chen C. Li Z. Chen Y. Zhang X. (2021). Polyphyllin Ⅲ-Induced Ferroptosis in MDA-MB-231 Triple-Negative Breast Cancer Cells Can Be Protected against by KLF4-Mediated Upregulation of xCT. Front. Pharmacol. 12, 670224. 10.3389/fphar.2021.670224 34040532 PMC8141818

[B134] Zhu M. Zhang H. Pedersen K. S. Foster N. R. Jaszewski B. L. Liu X. (2022). Understanding Suboptimal Response to Immune Checkpoint Inhibitors. Adv. Biol. (Weinh), e2101319. 10.1002/adbi.202101319 35343107

[B135] Zhu X. Fan W. G. Li D. P. Kung H. Lin M. C. (2011). Heme Oxygenase-1 System and Gastrointestinal Inflammation: a Short Review. World J Gastroenterol 17, 4283–4288. 10.3748/wjg.v17.i38.4283 22090784 PMC3214703

[B136] Zhu X. Fan W. G. Li D. P. Lin M. C. Kung H. (2010). Heme Oxygenase-1 System and Gastrointestinal Tumors. Wjg 16, 2633–2637. 10.3748/wjg.v16.i21.2633 20518085 PMC2880776

[B137] Zou Y. Palte M. J. Deik A. A. Li H. Eaton J. K. Wang W. (2019). A GPX4-dependent Cancer Cell State Underlies the Clear-Cell Morphology and Confers Sensitivity to Ferroptosis. Nat. Commun. 10, 1617. 10.1038/s41467-019-09277-9 30962421 PMC6453886

[B138] Zou Z. Tao T. Li H. Zhu X. (2020). mTOR Signaling Pathway and mTOR Inhibitors in Cancer: Progress and Challenges. Cell Biosci. 10, 31. 10.1186/s13578-020-00396-1 32175074 PMC7063815

